# General Inquiries

**Published:** 1886-10-02

**Authors:** 


### General Enquiries.

### The Cost of Nursing.

"Will any of your readers oblige by referring me to a source of information regarding the cost of nursing, apart from that of other maintenance charges, in the large general hospitals of the metropolis and of the provinces?"

*** We have made arrangements for the publication of statistics, giving the desired information, in an early number of this journal.

### Provident Dispensaries and Out-Patients' Departments.

A. B. C. would like to know any case where the Provident Dispensary System has been adopted by the out-patient department of a general hospital, and with what result.

*** The Provident Dispensary System has been adopted by the authorities of the Royal Albert Hospital, Devonport, and has proved in every way satisfactory and successful.

